# Growth Differentiation Factor-15 Is Considered a Predictive Biomarker of Long COVID in Non-hospitalized Patients

**DOI:** 10.7759/cureus.59433

**Published:** 2024-05-01

**Authors:** Rie Ono, Shin Takayama, Michiaki Abe, Ryutaro Arita, Takaaki Abe, Tadashi Ishii

**Affiliations:** 1 Department of Kampo Medicine, Tohoku University Hospital, Sendai, JPN; 2 Department of Education and Support for Regional Medicine, Tohoku University Hospital, Sendai, JPN; 3 Department of Anesthesiology and Perioperative Medicine, Tohoku University Hospital, Sendai, JPN; 4 Department of Kampo and Integrative Medicine, Tohoku University Graduate School of Medicine, Sendai, JPN; 5 Department of Clinical Biology and Hormonal Regulation, Tohoku University Graduate School of Medicine, Sendai, JPN; 6 Department of Nephrology, Endocrinology, and Vascular Medicine, Tohoku University Graduate School of Medicine, Sendai, JPN

**Keywords:** fibroblast growth factor-21, mitokine, non-hospitalized, biomarker, mitochondria, growth/differentiation factor 15, long covid

## Abstract

Mitochondrial dysfunction is associated with various diseases. Mitochondria plays a regulatory role during infection. The association between mitokines and subsequent COVID progression has not been previously studied. The retrospective cohort study aimed to investigate the potential of serum mitokines as long COVID biomarkers in non-hospitalized patients. Patients with confirmed SARS-CoV-2 infection and blood test reports between January 2021 and April 2023 were included. Patients were categorized into two groups, the recovered and long COVID groups, based on fatigue, decline in focus, and pain. Serum levels of growth differentiation factor 15 (GDF-15) and fibroblast growth factor-21 (FGF-21), which are affected by mitochondrial function, along with inflammatory and vascular endothelium markers, were measured using enzyme-linked immunosorbent assays (ELISA). A receiver operating characteristic curve was used to screen the biomarkers. The threshold value of GDF-15 in the acute phase was 965 pg/mL (sensitivity: 71.4%, specificity: 83.3%), indicating that GDF-15 may be associated with the presence of symptoms three months post onset. No association with inflammatory markers and vascular structures was observed. Therefore, elevated GDF-15 levels in the acute phase may act as a predictive biomarker of long COVID.

## Introduction

Some individuals infected with the severe acute respiratory syndrome coronavirus 2 (SARS-CoV-2) suffer from health issues known as "long COVID," which persists for four weeks or more after the initial infection phase [1]. Symptoms vary widely, with a high prevalence, and recent studies have shown that approximately 10% of individuals experience symptoms of long COVID at six months post infection [2]. While many gradually recover, some experience long-term impediments in their daily lives.

Biomarkers of long COVID aid in treatment and offer insights into the underlying pathophysiology. Previous studies showed that inflammatory markers, such as interleukin (IL)-6, C-reactive protein (CRP), and tumor necrosis factor (TNF)-alpha, may be a potential core set of biomarkers for long COVID [3-5]. However, that study included hospitalized patients. Therefore, inflammatory markers may reflect the overlapping pathophysiology of chronic crmitochondrial function due to its regulatory role itical care illness and post-intensive care syndrome. Long COVID also occurs in outpatients. We thought that the long COVID biomarkers needed to be estimated only in non-hospitalized patients.

This study investigated mitochondrial function due to its regulatory role in infection, inflammation, recovery, and cellular homeostasis [6]. Viral infections cause excessive production of mitochondrial reactive oxygen species, which disrupt the mitochondrial morphology and function [7]. Mitochondrial insufficiency is related to aging [8], immune dysfunction [9,10], and metabolic dysregulation [11]. The symptoms of long COVID can be explained by similarities to aging, immune dysfunction, and metabolic dysregulation. Mitochondrial insufficiency may be linked to the onset of long COVID. Mitokines, such as growth differentiation factor-15 (GDF-15) and fibroblast growth factor-21 (FGF-21), reflect mitochondrial dynamics. GDF-15 is associated with the severity and prognosis of acute COVID-19 [12,13]. However, no previous studies have shown an association between mitokines and COVID-19 progression into long COVID. Therefore, it is crucial to evaluate the association of mitokine dynamics with long COVID.

In this retrospective study, we aimed to investigate whether serum GDF-15 and FGF-21 may predict the onset and prognosis of long COVID in non-hospitalized patients with mild or moderate symptoms in the acute phase. This would help improve the outcomes of long COVID patients and help us understand the pathophysiology of long COVID.

## Materials and methods

Materials and methods

Isolation facilities dedicated to observing acute COVID-19 patients were present in Japan prior to May 7, 2023. Miyagi Prefecture established a facility that provided medical treatment based on patients’ conditions [14]. In this facility, we conducted a project under the Tohoku University Medical Megabank Organization to store blood samples from patients requiring medical management. This was done to explore the relationship between the symptoms and progression of COVID-19 and the corresponding blood data.

After receiving acute medical treatment, patients continued their care based on symptoms at the outpatient clinic of Tohoku University Hospital.

Study design and patient selection

This retrospective cohort study included patients who had medical treatment in the isolation facilities for COVID-19 in Miyagi Prefecture between January 2021 and April 2023 and needed medical checks for residual symptoms over three months post onset. The exclusion criteria were unconfirmed to SARS-CoV-2, requiring oxygenation, hospitalization, and pregnancy.

Written informed consent was obtained from all the participants before blood sampling. Information of included patients was collected, such as background, age, sex, height, body weight, body mass index, smoking, vaccination, severity of acute phase, and past medical history.

Patients who continued the medical treatment approximately three months post onset were examined regarding the appearance or deterioration of symptoms based on a visual analog scale (VAS), and blood tests were carried out. We categorized patients into two groups based on the VAS scores for fatigue, decline in focus, and pain: the recovered and long COVID groups. Patients in the recovered group were asymptomatic, with VAS scores for the aforementioned symptoms at zero, Conversely, patients in the long COVID group continued to experience any of these symptoms three months post onset. These symptoms were selected due to their frequent occurrence in long COVID cases and their impact on social functioning.

Sample processing

Blood samples were centrifuged and stored at -80 ℃ at the Tohoku University Medical Megabank Organization. We analyzed serum samples from the acute phase and approximately three months post onset. We measured GDF-15 and FGF-21 as indicators of mitochondrial function. Additionally, we identified several potential biomarkers for comparison with GDF-15 and FGF-21: inflammation markers IL-6, TNF-α, and IL-8; anti-inflammation marker IL-10; and endothelium markers vascular cell adhesion molecule-1 (VCAM-1) and syndecan-1.

They were measured using enzyme-linked immunosorbent assays (ELISAs) (GDF-15; MILLIPLEX® Human Cancer Metastasis Biomarker Magnetic Bead Panel, FGF-21; MILLIPLEX® Human Myokine Magnetic Bead Panel, IL-6, TNF-α, IL-8, IL-10, VCAM-1, syndecan-1; Luminex® Discovery Assay Human Premixed Multi-Analyte Kit). We also included in the analysis the total lymphocyte count and levels of lactate dehydrogenase (LDH), CRP, and D-dimer, which were measured in the laboratory of the outpatient at Tohoku University Hospital as part of routine medical care.

Ethical approval

The study was conducted in compliance with the Declarations of Helsinki and Tokyo for humans and approved by the ethical committee of Tohoku University (Miyagi, Japan) (approval number: 2022-1-801).

Statistical analysis

The Mann-Whitney U test was performed to determine statistically significant differences between the backgrounds and measured data of both groups in the acute phase and approximately three months after onset. Statistical significance was set at p < 0.05 (a two-tailed test). A receiver operating characteristic (ROC) curve in the acute phase was used to assess the performance of the measured data as a prediction, whereas the ROC after three months was used to assess the performance of diagnosing long COVID. Associations were evaluated using the area under the curve (AUC), and thresholds were calculated using the Youden index. An AUC ≥ 0.7 was considered indicative of predictive ability. Fisher's exact test was performed to evaluate whether the threshold was relevant to the development of long COVID, with p < 0.05 considered statistically significant. Statistical analyses were performed using GraphPad Prism (version 10.1.1; San Diego, CA).

## Results

Consent for the study and blood samples was obtained from 197 cases during the acute phase. Subsequently, blood samples from 13 cases (6.6%) were collected after three months. In 13 cases, seven cases (53.8%) were categorized into the long COVID group. Table 1 lists the background characteristics of the patients. The patient age ranged from 31 to 75 years, and eight patients (57.1%) were women. The number of vaccinations was noted for reference only, given the variab, with similar distributions observedility depending on the year of infection. There were no significant differences between the background factors of the two groups; however, five patients (71.4%) in the long COVID group were women.

**Table 1:** Patient information. *1: SpO_2_ ≥ 96%, no dyspnea, no signs of pneumonia; *2: 93% < SpO_2 _< 96%, dyspnea, signs of pneumonia, no need for oxygen administration

	All	Recovered	Long COVID	
	N=13	N=6	N=7	p
Age, years (range)	48 (31-75)	44.5 (31-75)	56 (33-69)	0.31
Sex, Female, N (%)	8 (57.1)	3 (50.0)	5 (71.4)	0.59
Body mass index kg/m^2^ (range)	22.0 (17.4-30.0)	25.0 (17.4-30.0)	21.7 (18.4-25.1)	0.15
Smoking, N (%)	4 (30.8) unknown^1^	2 (33.3) unkown^1^	2 (28.6)	>0.99
Vaccination, N (%)				
<3 times	5 (38.5)	3 (50.0)	2 (28.6)	0.59
3 times ≦	8 (61.5)	3 (50.0)	5 (71.4)	
Severity, N (%)				
Mild*^1^	5 (38.5)	3 (50.0)	2 (28.6)	0.59
Moderate I^＊2^	8 (61.5)	3 (50.0)	5 (71.4)	
Past medical history, N (%)				
Neurological diseases	0 (0.0)	0 (0.0)	0 (0.0)	-
Cardiovascular diseases	5 (38.5)	2 (33.3)	3 (42.9)	0.58
Respiratory diseases	4 (30.80	3 (50.0)	1 (14.3)	0.27
Diabetes mellitus	0 (0.0)	0 (0.0)	0 (0.0)	-
Hyperlipidemia	5 (38.5)	1 (16.7)	4 (57.1)	0.27
Mental disorders	0 (0.0)	0 (0.0)	0 (0.0)	-

**Figure 1:**
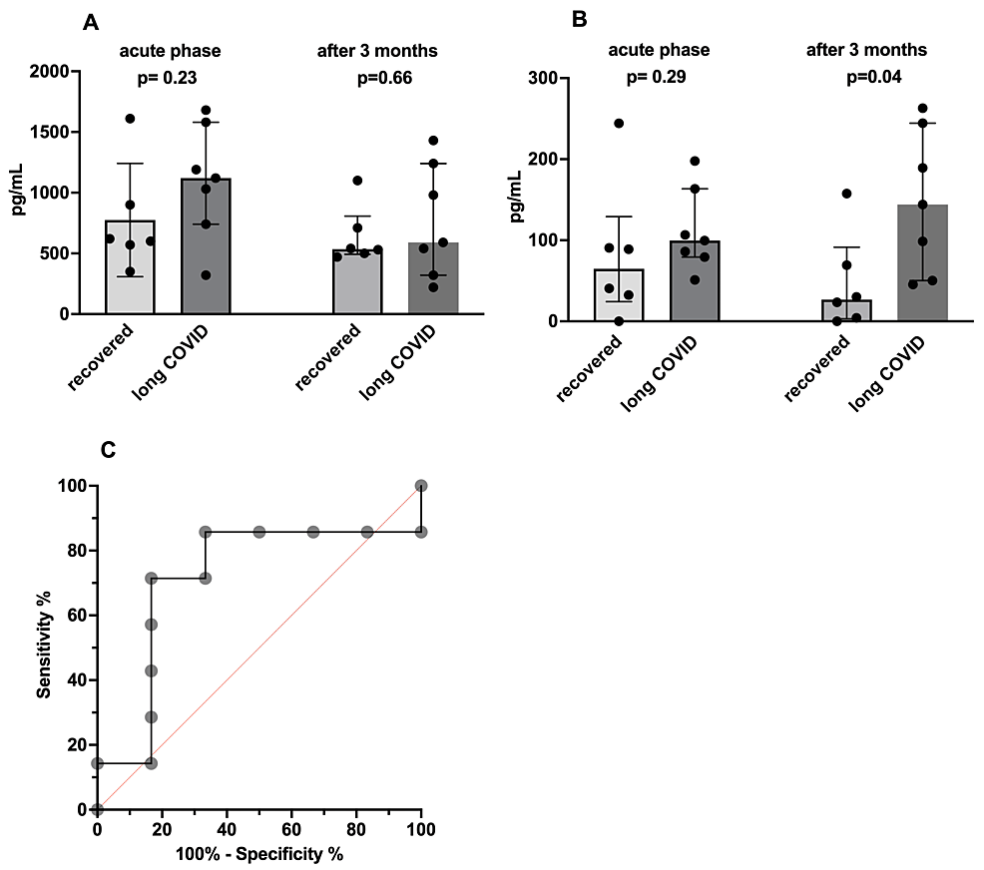
Measurements of serum levels of GDF-15 and FGF-21 with the ROC curve of the serum GDF-15 level. (A) Measurements of serum GDF-15 level: The two bar graphs on the left show serum GDF-15 levels in the acute phase of COVID-19. The two bar graphs on the right show serum GDF-15 levels after three months. On the left side of each is a graph of the measurement of the recovered group, and on the right side of each is a graph of the measurement of the long COVID group. (B) Measurements of serum FGF-21 level: The two bar graphs on the left show serum FGF-21 levels in the acute phase of COVID-19. The two bar graphs on the right show serum FGF-21 levels after three months. On the left side of each is a graph of the measurement of the recovered group, and on the right side of each is a graph of the measurement of the long COVID group. (C) Receiver operating characteristic (ROC) curve of serum GDF-15 level in the acute phase. GDF-15: growth/differentiation factor-15; FGF-21: fibroblast growth factor-21

Serum GDF-15 and FGF-21 levels are shown in Figures 1A, 1B. No significant difference was observed between the median acute phase GDF-15 level (610 pg/mL; range: 320-1,610 pg/mL) of the recovered group and that (1,120 pg/mL; range: 320-1,680 pg/mL) of the long COVID group (p = 0.23). However, there was variation in the distribution of values between the two groups during the acute phase. The AUC of the ROC curve (Figure 1C) was 0.71, indicating a moderate predictive ability. The threshold value was 965 pg/mL (sensitivity: 71.4%, specificity: 83.3%). Of the patients with GDF-15 levels of 965 pg/mL or higher, five out of six (83.3%) exhibited symptoms, compared to two out of seven (28.6%) with levels below 965 pg/mL. Although not statistically significant (Fisher's exact test, p = 0.08), many patients with serum GDF-15 levels of 965 pg/mL or higher experienced symptoms. After three months, serum GDF-15 levels were 535 pg/mL (range: 470-1,100) in the recovered group and 590 pg/mL (range: 220-1,430) in the long COVID group (p = 0.66).

Serum FGF-21 levels in the acute phase had a median of 64.7 pg/mL (range: 0-244.3 pg/mL) and 99.6 pg/mL (range: 51.5-197.8 pg/mL) in the recovered and long COVID groups, respectively, with no significant difference observed (p = 0.29). The AUC of the ROC curve was 0.69, indicating poor predictive ability. However, after three months, serum FGF-21 levels were 26.8 pg/mL (range: 0-157.6 pg/mL) in the recovered group and 117.4 pg/mL (range: 45.4-244.4 pg/mL) in the long COVID group (p = 0.04), demonstrating significantly higher levels in the long COVID group. The AUC of the ROC curve was 0.86, suggesting good diagnostic ability. Nevertheless, the threshold value was 37.8 pg/mL (with a sensitivity of 100% and a specificity of 66.7%), which is not unusually high.

The values of inflammatory and vascular endothelial markers are shown in Table 2, including the median values (range) and statistical results for both the recovered and long COVID groups. In the acute phase, IL-6 levels were 1.9 (0.0-8.4) pg/mL in the recovered group and 0.0 (0.0-6.9) pg/mL in the long COVID group (p = 0.34). TNF-α levels were 4.5 (1.4-5.5) pg/mL and 3.1 (2.6-5.3) pg/mL, respectively (p = 0.70); IL-8 levels were 9.7 (5.4-15.7) pg/mL and 10.9 (2.3-28.3) pg/mL, respectively (p = 0.57); IL-10 levels were 4.1 (0.0-10.0) pg/mL and 7.7 (0.0-14.1) pg/mL, respectively (p = 0.26); LDH levels were 160 (144-170) U/L and 166 (153-201) U/L, respectively (p = 0.19); CRP levels were 2.2 (0.2-6.8) mg/dL and 1.3 (0.1-7.7) mg/dL, respectively (p = 0.45); lymphocyte count was 1,305 (720-1,580) and 1,170 (640-1,950)/µL, respectively (p = 0.95); vascular cell adhesion molecule-1 (VCAM-1) levels were 1,940 (656.7-4,237.4) ng/mL and 1,215 (564.6-4,317.3) ng/mL, respectively (p = 0.73); syndecan-1 levels were 2.7 (2.4-4.5) ng/mL and 3.2 (2.4-4.4) ng/mL, respectively (p = 0.39); and D-dimer levels were 0.13 (0.10-0.26) and 0.15 (0.11-0.24) µg/mL, respectively (p = 0.81). These measurements showed no significant differences between the two groups, with similar distributions observed.

**Table 2:** The levels of markers, excluding GDF-15 and FGF-21. IL: interleukin, TNF-α: tumor necrosis factor-α, LDH; lactate dehydrogenase, CRP; C-reactive protein, VCAM-1; vascular cell adhesion molecule

Markers	Acute phase			After 3 months		
Median values (range)	Recovered group	Long COVID group	p	Recovered group	Long COVID group	p
IL-6 (pg/µL)	1.9 (0.0–8.4)	0.0 (0.0–6.9)	0.34	0.17 (0.0–0.6)	0.0 (0.0–0.62)	0.81
TNF-α (pg/µL)	4.5 (1.4–5.5)	3.1 (2.6–5.3)	0.70	3.0 (1.7–3.6)	2.2 (1.6–4.4)	0.22
IL-8 (pg/µL)	9.7 (5.4–15.7)	10.9 (2.3–28.3)	0.57	8.6 (2.4–18.3)	8.8 (5.4–11.4)	0.94
IL-10 (pg/µL)	4.1 (0.0–10.0)	7.7 (0.0–14.1)	0.26	4.1 (0.0–10.0)	7.7 (0.0–14.1)	0.60
LDH (U/L)	160 (144–170)	166 (153–201)	0.19	No data		
CRP (mg/dL)	2.2 (0.2–6.8)	1.3 (0.1–7.7)	0.45	0.06 (0.04–0.13)	0.04 (0.01–0.56)	0.45
Lymphocyte count (/µL)	1305 (720–1580)	1170 (640–1950)	0.95	No data		
VCAM-1 (ng/µL)	1940 (656.7–4237.4)	1215 (564.6–4317.3)	0.73	693.4 (528.4–1138.5)	854.3 (357.4–1248.7)	0.73
Syndecan-1 (ng/µL)	2.7 (2.4–4.5)	3.2 (2.4–4.4)	0.39	2.9 (2.2–4.6)	3.9 (2.5–4.7)	0.53
D-dimer (ug/mL)	0.13 (0.10–0.26)	0.11–0.24	0.81	No data		

After three months, the IL-6 levels were 0.17 (0.0-0.6) pg/mL in the recovered group and 0.0 (0.0-0.62) pg/mL in the long COVID group (p = 0.81); TNF-α levels were 3.0 (1.7-3.6) pg/mL and 2.2 (1.6-4.4) pg/mL, respectively (p = 0.22); IL-8 levels were 8.6 (2.4-18.3) pg/mL and 8.8 (5.4-11.4) pg/mL, respectively (p = 0.94); IL-10 levels were 4.1 (0.0-10.0) pg/mL and 7.7 (0.0-14.1) pg/mL, respectively (p = 0.60); CRP levels were 0.06 (0.04-0.13) mg/dL and 0.04 (0.01-0.56) mg/dL, respectively (p = 0.45); VCAM-1 levels were 693.4 (528.4-1,138.5) ng/mL and 854.3 (357.4-1248.7) ng/mL, respectively (p = 0.73); and syndecan-1 levels were 2.9 (2.2-4.6) ng/mL and 3.9 (2.5-4.7) ng/mL, respectively (p = 0.53). These measurements showed no significant differences between the two groups, with similar distributions observed. LDH and D-dimer levels, as well as the lymphocyte count, were not measured after three months.

## Discussion

Elevated levels of serum GDF-15 in the acute phase of COVID-19 were associated with the presence of symptoms, including fatigue, decline in focus, and pain, in non-hospitalized patients three months after infection in the present study. The elevated levels of serum GDF-15 in the acute phase of COVID-19 may predict the onset of long COVID in non-hospitalized patients. Mitochondria play a crucial role in maintaining individual homeostasis and dynamically altering metabolic and inflammatory responses throughout the body [10]. Therefore, levels of activated mitokines may provide a comprehensive assessment of the systemic status. Elevated serum GDF-15 levels are reportedly associated with undesirable aging and poor prognoses in chronic diseases and infections [15,16]. In this study, elevated serum GDF-15 levels suggested that mitochondrial modulation in the acute phase may influence the subsequent course of COVID-19. However, the serum GDF-15 level after three months was not associated with the presence of symptoms; therefore, its diagnostic utility may be low. GDF-15 exerts tissue-protective effects, where maintaining a good balance is crucial for stress response [17,18]. After three months, a transitional period of recovery from COVID-19 may ensue.

Serum FGF-21 levels in patients with long COVID were significantly higher after three months, but not beyond the physiological range. It is still unclear whether high levels of serum FGF-21 are beneficial or harmful [19]. Although FGF-21 may serve as a diagnostic marker for long COVID, further investigations are required.

Mitochondrial dysfunction is considered one of the mechanisms of long COVID [20], although it is not fully understood. Studies on the pathophysiology suggest an insufficient oxygen supply, a reduced rate of glucose usage, and immunological mechanisms from damaged mitochondria contribute to the condition. Long COVID is often compared to myalgic encephalomyelitis/chronic fatigue syndrome (ME/CFS) [21]. Patients with ME/CFS exhibit mitochondrial dysfunction or decreased adenosine triphosphate (ATP) production in skeletal muscle, cardiac muscle, and peripheral mononuclear blood cells [22]. Impaired mitochondrial function in skeletal muscle cannot meet increased ATP demand during activity, leading to fatigue. Moreover, glucose metabolism disturbances in the brain are associated with aging and cognitive impairment [23]. However, our study found no difference in serum levels of GDF-15 or FGF-21 between the recovered group and the long COVID group three months post onset. While mitochondrial function may recover in the chronic phase, the exact local conditions remain unclear. Additionally, reduced clearance of damaged mitochondria leads to neurodegenerative diseases [24] and activates the inflammasome associated with mitochondrial dysfunction, causing neuroinflammation [25]. These findings suggest that degenerated mitochondria from COVID-19 are not adequately removed, perpetuating neuroinflammation, which may induce symptoms of long COVID such as fatigue, decreased concentration, and pain. The results of this study indicated that inflammatory markers were not associated with long COVID. Previous studies have reported that inflammatory markers were predictors of the poor prognosis of COVID-19 [26] and useful as a biomarker of long COVID [27]. These studies included hospitalized patients who may have experienced symptoms similar to post-intensive care syndrome. This study included only non-hospitalized patients, whose inflammatory markers were not observed to be significantly elevated in the acute phase or after three months. Therefore, inflammatory markers cannot serve as biomarkers of long COVID in non-hospitalized patients.

The expression of the vascular endothelium marker, serum VCAM-1, was high in both the acute and chronic phases. SARS-CoV-2 infects via the angiotensin-converting enzyme-2 receptor, which is highly expressed in the vascular endothelium. COVID-19 is reportedly associated with microcirculation dysfunction and the destruction of the endothelial structure [28]. Long COVID, seen in patients who recovered from mild-to-moderate COVID-19, may be associated with subclinical multi-organ effects related to thrombosis [29]. The vascular endothelium markers may be associated with symptoms other than those defined as long COVID in this study.

This study has some limitations. Serum GDF-15 and FGF-21 levels are affected by age, which is also a long-term risk factor for COVID; therefore, age could have interfered with the conclusions regarding the potential of mitokines as biomarkers. The small sample size posed another limitation. This study was conducted using stored samples, thus limiting the number of samples available.

In this study, blood samples were collected from 197 patients during the acute phase, but only 13 patients required medical treatment for more than three months. In other words, the incidence of patients with residual symptoms was 6.7%, resulting in a small sample size for this study.

Furthermore, the status beyond three months of onset was not studied. These aspects should be considered in future studies.

## Conclusions

This study measured serum levels of GDF-15 and FGF-21, along with inflammatory and vascular endothelium markers. Elevated serum GDF-15 levels in the acute phase of COVID-19 may act as a predictive biomarker with the onset of long COVID, which enables early identification and management, thereby improving outcome. Additionally, this study provides valuable insights into the pathophysiology of long COVID.
